# Untargeted Mass Spectrometry Lipidomics identifies correlation between serum sphingomyelins and plasma cholesterol

**DOI:** 10.1186/s12944-018-0948-5

**Published:** 2019-02-02

**Authors:** Pierre Zalloua, Hanane Kadar, Essa Hariri, Layal Abi Farraj, Francois Brial, Lyamine Hedjazi, Aurelie Le Lay, Alexandre Colleu, Justine Dubus, David Touboul, Fumihiko Matsuda, Mark Lathrop, Jeremy K. Nicholson, Marc-Emmanuel Dumas, Dominique Gauguier

**Affiliations:** 1 Lebanese American University, School of Medicine, Beirut, Lebanon; 2 University Paris Descartes, 15 rue de l’Ecole de Médecine, 75006 Paris, France; 3 Cordeliers Research Centre, INSERM UMRS 1138, SorbonneUniversity, 15 rue de l’école de médecine, 75006 Paris, France; 4 Institute of Cardiometabolism and Nutrition, University Pierre & Marie Curie, 91 boulevard de l’Hôpital, 75013 Paris, France; 5 Institut de Chimie des Substances Naturelles, UPR2301, CNRS, Avenue de la Terrasse, 91198 Gif-sur-Yvette, France; 6 Center for Genomic Medicine, Kyoto University Graduate School of Medicine, Kyoto, Japan; 7 McGill University and Genome Quebec Innovation Centre, 740 Doctor Penfield Avenue, Montreal, QC H3A 0G1 Canada; 8 Computational and Systems Medicine, Department of Surgery and Cancer, Faculty of Medicine, Imperial College London, London, SW7 2AZ UK; 9 Lebanese American University, Box 36, Byblos, PO Lebanon

**Keywords:** Lipidomics, Lipoproteins, HDL cholesterol, Sphingolipids, UPLC-MS

## Abstract

**Background:**

Lipoproteins are major players in the development and progression of atherosclerotic plaques leading to coronary stenosis and myocardial infarction. Epidemiological, genetic and experimental observations have implicated the association of sphingolipids and intermediates of sphingolipid synthesis in atherosclerosis. We aimed to investigate relationships between quantitative changes in serum sphingolipids, the regulation of the metabolism of lipoproteins (LDL, HDL), and endophenotypes of coronary artery disease (CAD).

**Methods:**

We carried out untargeted liquid chromatography – mass spectrometry (UPLC-MS) lipidomics of serum samples of subjects belonging to a cross-sectional study and recruited on the basis of absence or presence of angiographically-defined CAD, and extensively characterized for clinical and biochemical phenotypes.

**Results:**

Among the 2998 spectral features detected in the serum samples, 1328 metabolic features were significantly correlated with at least one of the clinical or biochemical phenotypes measured in the cohort. We found evidence of significant associations between 34 metabolite signals, corresponding to a set of sphingomyelins, and serum HDL cholesterol. Many of these metabolite associations were also observed with serum LDL and total cholesterol levels but not as much with serum triglycerides.

**Conclusion:**

Among patients with CAD, sphingolipids in the form of sphingomyelins are directly correlated with serum levels of lipoproteins and total cholesterol. Results from this study support the fundamental role of sphingolipids in modulating lipid serum levels, highlighting the importance to identify novel targets in the sphingolipid metabolic pathway for anti-atherogenic therapies.

**Electronic supplementary material:**

The online version of this article (10.1186/s12944-018-0948-5) contains supplementary material, which is available to authorized users.

## Introduction

Coronary artery disease (CAD) is the leading cause of mortality caused by a complex interplay of genetic and environmental factors [1]. Accumulation of fatty deposits in the intima layer of arteries leads to arterial stiffness and formation of plaques that result in narrowing of arteries and eventually coronary artery stenosis [2]. Among all genetically determined intermediates mediating CAD risk phenotypes, elevated plasma LDL-cholesterol level is highly associated with CAD given its pivotal role in atherosclerosis and myocardial infarcts [3], whereas elevated plasma HDL plays a protective role against CAD [4].

Better understanding of molecular mechanisms that contribute to the regulation of cholesterol metabolism is needed to discover more biomarkers and therapeutic targets of CAD [5]. Functional genomics is one tool that can help with the identification of regulatory elements and their biological pathways related to cholesterol metabolism. Metabolomics and lipidomics are also powerful strategies to qualitatively and quantitatively analyze a wide range of small molecules in a biological sample, which represent endpoints of genome expression [6]. They provide a source of molecular endophenotypes (i.e. intermediate phenotypes) that can massively refine phenotypic information in patients and healthy individuals [7]. Their regulation integrates the combined effects of genetics and environmental exposures (e.g. microbiota, lifestyle) on health [8], and they provide great potential in biomarker discovery that can ultimately lead to the implementation of precision medicine strategies [9].

Metabolome-wide association studies (MWAS) have been proposed to predict disease condition [10, 11] and have led to the identification of biomarkers associated with risk factors of chronic diseases such as hypertension [12] and obesity [13]. In this study, we applied an untargeted lipidomic profiling strategy based on ultra-performance liquid chromatography coupled to mass spectrometry (UPLC-MS) to identify metabolic features associated with CAD risk factors and changes in clinical and intermediate biochemical phenotypes in patients screened for evidence of CAD. The 2998 spectral features detected in the serum samples were used to identify significant correlations between metabolic features and clinical or biochemical phenotypes measured in our study subjects, including specific correlations between sphingomyelins and serum HDL cholesterol, which were largely independent of serum triglycerides and CAD.

## Materials and methods

### Study subjects

A total of 109 subjects aged 17–81 (73 males and 36 females) recruited between 2006 and 2009 for inclusion in the FGENTCARD patient collection [14] were selected for this study on the basis of the presence (for cases) or absence (for controls) of coronary artery stenosis. These subjects were originally referred to a catheterization care unit for clinical evaluation. The 4 main coronary arteries (left main artery, left anterior descending artery, left circumflex artery, right coronary artery) were analyzed by angiography. All individuals were assessed for presence of coronary stenosis following angiography analysis. For CAD phenotypes, cases were patients with ≥50% stenosis in any of the coronaries, and controls were subjects with < 50% stenosis in any of the coronaries. Cardiologists performing the coronary angiography collected a 20 mL blood sample from the peripheral femoral artery of 12 h-fasting patients for serum preparation.

Data on the socio-demographic background of the patients were recorded by trained healthcare professionals. Annotations were coded from medical charts according to our study protocol, which included results from laboratory tests, prescribed medications, and presence of co-morbid disease conditions (diabetes, hypertension, hyperlipidemia, and obesity). Patients were classified hypertensives based on physician diagnosis with documented treatment in their medical charts by one of the following antihypertensive agents: beta blockers, diuretics, calcium antagonists, angiotensin-converting enzyme inhibitors, angiotensin receptors inhibitors, and central antihypertensive agents. Diagnosis if T2DM and hyperlipidemia were based on physicians’ information in the patients charts and were confirmed by regular intake of antidiabetic and cholesterol-lowering drugs, respectively. Blood HDL, LDL, triglyceride, and glucose levels were recorded. Patients’ Height and weight were also recorded, and their BMI derived.

The study protocol conforms to the ethical guidelines of the 1975 Declaration of Helsinki. Patients provided a written consent for the study, and the study protocol was approved by the Institutional Review Board (IRB) at the Lebanese American University.

### Ultra-performance liquid chromatography coupled to high-resolution mass spectrometry

Sample analysis was carried out with a Waters Acquity UPLC® (Waters Corp, Saint-Quentin en Yvelines, France) fitted with an Acquity CSH C18 column (2.1 × 100 mm, 1.7 μm) and a corresponding guard column (Acquity CSH 1.7 μM) to analyse lipids. Oven temperature and flow rate were consistently at 55 °C and 0.4 ml/min, respectively, for a volume of injection of 5 μl. The total run time was 24 min. The mobile phase consisted of solvent A (0.1% formic acid and ammonium formate in a mix of water (40%) and acetonitrile (60%) and solvent B (0.1% formic acid and ammonium formate in a mix of water (10%) and acetonitrile (90%). A binary gradient consisted of above described mobile phases A and B as recommended by Waters. At the end of the gradient mobile phase B was maintained at 99% for 4 min in order to wash the column and avoid sample carry-over.

The UPLC system was coupled with a Q-Exactive™ hydrid quadrupole-Orbitrap mass spectrometer (Thermo Fisher Scientific, Illkirch, France). Instrument calibration was performed by infusing a calibration mixture (caffeine, MRFA and Ultramark® 1621). A heated-electrospray ionization (HESI-II, Thermo Fisher Scientific, Illkirch, France) interface was used with the following parameters: S-Lens 50 V, Sheat gas: 65, Auxiliary gas: 25 arbitrary units, capillary voltage 3 kV, capillary temperature 350 °C and vaporization temperature 60 °C.The autogain control (AGC) parameter was defined as 3e6 ions and the maximum injection time was set to 200 ms. Full scan was acquired in positive and negative ion modes simultaneously with a resolution of 70,000 full width at half maximum (FWHM), in the scan range of *m/z* 85–1275.

### Untargeted Metabolomic data analysis

MS data acquired from UPLC-MS were analyzed with standard protocols and *food and drug administration* (FDA) guidelines [15, 16]. The preprocessing steps of MS data, including peak picking, peak grouping, retention time correction and annotation of isotopes and adducts, were performed using XCMS and CAMERA tools implemented in R statistical language (v 3.1.0) (http://www.bioconductor.org). Raw LC-MS files were initially processed to extract profiles of positive and negative ionization modes separately and then converted into mz XML format to be preprocessed directly by the XCMS and CAMERA tools. The wavelet-based peak picking approach (centwave) was used for the identification of Regions Of Interest (ROI). Preprocessing of MS data resulted in a peak table in which each metabolomics spectral feature was characterized by a retention time (RT), mass to charge ratio (m/z), an intensity estimate determined by the area under the peak and the annotation attributed by CAMERA.

Several filters were then applied on the resulting matrix in order to reduce the size of the data matrix by retaining only consistent spectral features in the individuals of the cohort. First, peaks with more than 40% of missing values were discarded. Next, a quality control (QC) strategy was applied to assess the performance of the analytical process and ensure that data quality meets FDA acceptance criteria [17]. The QC strategy was based on the use of a pooled QC sample, which was injected every 10 samples throughout the analytical run to detect deviations in signal related to instrumental drift, and therefore to assess the suitable quality of data. The retained features were first normalized based on a median fold change normalization approach [18]. A threshold of 30% was set for relative standard deviation (RSD) calculated for each metabolic feature in the QC samples, which is an accepted standard to assess consistency in metabolomic studies [15, 16]. Finally, the resulting matrix was used for multivariate and univariate statistical analysis (principal component analysis and linear regression). m/z information of significantly associated features was used to search the online human metabolome database (HMDB, http://www.hmdb.ca) for metabolite annotation. A candidate metabolite and a metabolic feature were matched when a mass difference was less than 1 ppm.

### Metabolite attributions of UPLC-MS features

To precisely confirm compound annotations, MS/MS experiments were performed on a Q-Exactive and a Q-TOF instrument. The Q-TOF instrument was also used to determine acyl anions corresponding to fatty acid moieties. To identify lipid compounds, MS/MS experiments were performed at 3 collision energies of 10, 20 and 30 eV, combined at the end the complete spectrum. This method allowed us to obtain both unfragmented and fragmented signals. This “shotgun” LC-MS approach relied on specific detection of [M + H]^+^ and [M-H]^−^, as well as adducts [M + NH_4_]^+^ and [M + HCOO]^−^. Distinct classes of lipids, including fatty acids, glycerophospholipids and sphingolipids could be detected. For each class of lipids, a specific fragmentation pattern was observed as previously reported [19].

### Statistical analyses

Statistical analysis of untargeted metabolomic data was performed to test the association of each metabolic feature with continuous phenotypes (triglycerides, HDL, LDL, total cholesterol and fasting plasma glucose (FBS) using linear regression models, and with CAD using logistic regression models. Normality assumption of the residuals of each metabolic feature was investigated by Shapiro-Wilk test. The SPSS statistical software was used then to perform the statistical analyses and determine statistical significance for *p*-values < 0.05. False discovery rates (FDR) were corrected using the Benjamini-Hochberg method to adjust p-values for false discovery involving multiple comparisons.

## Results

### Clinical and biochemical data analysis

The study population consisted of 109 individuals with a mean age of 53.38 ± 1.07 years (range 17–81 years), a mean body weight of 77.59 ± 1.56 kg, a mean body mass index (BMI) of 27.77 ± 0.47 kg/m^2^, a mean total cholesterol of 189.91 ± 3.74 mg/dL, a mean LDL of 116.52 ± 3.13 mg/dL, a mean HDL-C level of 39.94 ± 0.98 mg/dL and a mean fasting plasma glucose of 107.81 ± 3.96 mg/dL (Table 1). Patients with coronary stenosis were significantly older than controls. Family history of cardiac diseases was more present in cases than in controls, most prominently in males. Plasma concentrations of total and LDL cholesterol were significantly more elevated in male cases than in male controls.

**Table 1:** Demographic, clinical and biochemical features of cases with coronary artery stenosis and controls, whose sera were used for lipidomic analyses. Data are means ± SEM. Number of individuals is reported in parentheses

	Total	Controls	Cases	Male controls	Male Cases	Female controls	Female Cases
Demographics							
Age (y)	53.38 ± 1.07	53.66 ± 1.6 (62)	53 ± 1.3 (47)	52.1 ± 2.3 (36)	52.5 ± 1.3 (37)	55.8 ± 2.1 (26)	54.9 ± 3.9 (10)
Type 2 diabetes (Number, %)	21 (19.27)	10 (16.12)	9 (19.14)	4 (11.11)	6 (16.21)	4 (15.38)	3 (30)
Hyperlipidemia (Number, %)	20 (18.35)	10 (16.12)	8 (17.02)	4 (11.11)	6 (16.21)	4 (15.38)	2 (20)
Hypertension (Number, %)	22 (20.18)	10 (16.12)	12 (25.35)	6 (16.66)	9 (24.32)	4 (15.38)	3 (30)
Anthropometric variables							
Body weight (kg)	77.6 ± 1.6	74.5 ± 2.1 (62)	81.6 ± 2.1 (47)	79.5 ± 2.7 (36)	84.4 ± 2.2 (37)	67.5 ± 2.8 (26)	71.6 ± 4.5 (10)
Body mass index (kg/m^2^)	27.8 ± 0.5	27.1 ± 0.6 (62)	28.6 ± 0.7 (46)	27 ± 0.8 (36)	28.7 ± 0.7 (37)	27.3 ± 0.9 (26)	28.2 ± 2.2 (9)
Metabolic and inflammatory variables							
Plasma glucose (mg/dL)	107.81 ± 3.96	99.04 ± 2.3 (49)	117.5 ± 7.7 (44)	100.9 ± 3.7 (27)	115.1 ± 8.2 (35)	96.7 ± 2.5 (22)	127.3 ± 21.5 (9)
Total cholesterol (mg/dL)	189.91 ± 3.74	185.9 ± 4.9 (59)	195.1 ± 5.6 (45)	185 ± 3.7 (33)	200.1 ± 5.6 (35)	187.1 ± 8.3 (26)	177.7 ± 15.2 (10)
HDL cholesterol (mg/dL)	39.94 ± 0.98	41.2 ± 1.4 (59)	38.3 ± 1.2 (46)	38.6 ± 1.6 (33)	38.1 ± 1.4 (36)	44.5 ± 2.5 (26)	39 ± 2.9 (10)
LDL cholesterol (mg/dL)	116.52 ± 3.13	111.9 ± 3.9 (59)	122.4 ± 4.9 (46)	112.3 ± 4.9 (33)	126.1 ± 5.4 (36)	111.4 ± 6.4 (26)	108.9 ± 12.0 (10)
Triglycerides (mg/dL)	196.91 ± 11.44	187.5 ± 15.5 (59)	208.9 ± 17.1 (46)	206.2 ± 22.3 (33)	218.9 ± 20.7 (36)	163.7 ± 20.4 (26)	173.3 ± 23.6 (10)
Family history							
Cardiac disease	81 (74.31)	39 (62.90)	42 (89.36)	18 (50)	33 (89.18)	21 (80.76)	9 (90)
Hypertension	73 (66.97)	42 (67.74)	31 (65.95)	25 (69.44)	23 (62.16)	17 (65.38)	8 (80)
Type 2 diabetes	63 (57.80)	35 (56.45)	27 (57.44)	24 (66.66	20 (54.05)	11 (42.30)	7 (70)

The main demographic and biochemical features of the individuals, whose sera were used for metabolomics analyses, are also shown in Table 1. Plasma glucose and cholesterol levels and body mass index were not significantly different in males and females. The majority of participants were males (67%), non-diabetic (81%), and with strong family history of cardiac disease (74%); 22% of the subjects were hypertensive and 43% showed evidence of coronary artery stenosis. There were no significant gender differences in disease or family history of disease.

### Analysis of UPLC-MS data

To identify metabolites associated with clinical phenotypes and plasma lipoprotein levels, we carried out untargeted lipidomic analyses. We were able to capture spectral signals corresponding to a wide range of lipids as illustrated by the total ion current chromatogram profiles obtained with a CSH C18 column, which shows the retention time windows of lipid classes in the UPLC chromatograms in positive and negative ion modes (Additional file 1: Figure S1). There were higher numbers of compounds detected in positive mode than in negative mode.

Following preprocessing and filtering steps, lipidome profiling with the CSH C18 column identified 2998 features that met the acceptance criterion (i.e. RSD < 30%), including 1601 (89%) on the negative ionization mode and 1397 (92%) on the positive ionization mode. Multivariate PCA analysis demonstrated the absence of technical drift during data acquisition process as illustrated by the PCA scores plot representation of QC samples in both ionization modes with the CSH C18 column (Additional file 1: Figure S2). Metabolomics data from QC samples were tightly clustered, which demonstrates an acceptable reproducibility of the retained set of metabolic features as well as a good stability of UPLC-MS profiling measures.

### Association of UPLC-MS features with clinical and biochemical phenotypes

After adjusting for age, sex and the intake of medications by patients prior to serum samples analysis, we found a significant association (FDR-adjusted *p* < 0.05) between 1329 spectral features, including 678 in negative ionization mode and 651 in positive mode, and at least one phenotype (Additional file 2: Table S1).

**Fig. 1:**
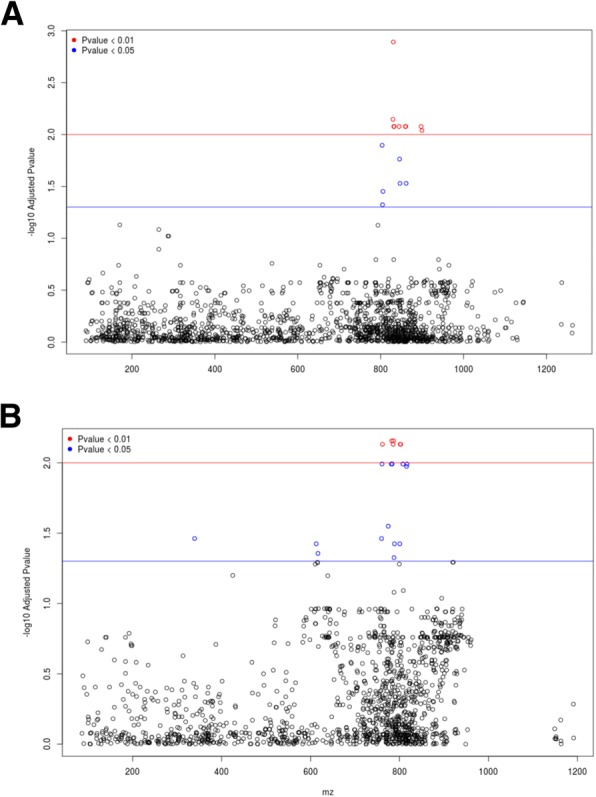
Untargeted metabolomic-wide association between metabolic features and HDL cholesterol. Metabolomic data were obtained by liquid chromatography mass spectrometry (UPLC-MS) with a CSH C18 column. Quantitative serum metabolomic data were tested for correlation with plasma HDL cholesterol (A,B). Spectral features referenced by their mass to charge ratio (X-axis) are plotted against the statistical significance of the association to body mass index (Y-axis). Association results are shown for data in negative (**a**) and positive (**b**) ionization modes. Details of the spectral signals (mass to charge ratio and retention time), correlations statistics, along with co-associations with other phenotypes are given in Table 2 and Additional file 2: Table S1

HDL cholesterol was significantly associated with 34 lipid features in positive (*n* = 19) and negative (*n* = 15) ionization modes (Fig. 1a and b). A large proportion of these features (*n* = 27) were also significantly associated with elevated serum total and LDL cholesterol (Table 2). Total and LDL cholesterol showed much denser and more complex patterns of association to lipidomic data than that of HDL cholesterol (Additional file 1: Figure S3), consisting of 476 (Total cholesterol) and 291 (LDL cholesterol) features, including 262 common for both total and LDL cholesterol (Additional file 2: Table S1). The patterns of association of lipidomic data to serum triglycerides was also complex and involved a total of 1020 associations, including 179 which were common with total cholesterol.

**Table 2:** Details of metabolic features significantly associated (FDR adjusted P < 0.05) with quantitative variations of HDL cholesterol, and relationships with other phenotypes analyzed in the study cohort. Correlations statistics of metabolic features are given. m/z, mass to charge ratio. rt., retention time. Tchol, Total cholesterol. TGs, Triacylglycerols. Corr, Correlation. Ns, not statistically significant

Metabolic Features				HDL		LDL		TGs		Tchol		FBS	
Ionisation Mode	mz	rt	Isotopes	Adjusted P	Corr	Adjusted P	Corr	Adjusted P	Corr	Adjusted P	Corr	Adjusted P	Corr
Negative	803.628	636.426	[176] [M]-	0.001	0.337	1.65 × 10^− 4^	0.394	Ns	–	1.85 × 10^− 4^	0.392	Ns	–
Negative	804.632	636.426	[176] [M + 1]-	0.001	0.292	1.06 × 10^−4^	0.411	Ns	–	1.85 × 10^− 4^	0.390	Ns	–
Negative	805.635	636.317	[176] [M + 2]-	0.001	0.304	1.65 × 10^−4^	0.404	Ns	–	1.85 × 10^− 4^	0.402	Ns	–
Negative	829.644	662.705	[209] [M]-	1.99 × 10^− 4^	0.368	0.002	0.311	Ns	–	0.002	0.308	Ns	–
Negative	830.647	662.659	[209] [M + 1]-	1.90 × 10^− 4^	0.400	0.002	0.320	0.030	−0.221	0.002	0.310	Ns	–
Negative	831.66	783.461	[211] [M]-	2.61 × 10^−4^	0.282	3.07 × 10^− 4^	0.380	Ns	–	1.85 × 10^− 4^	0.390	Ns	–
Negative	832.663	783.459	[211] [M + 1]-	2.61 × 10^− 4^	0.281	2.67 × 10^− 4^	0.383	Ns	–	1.85 × 10^− 4^	0.393	Ns	–
Negative	844.663	761.148	[231] [M + 1]-	1.99 × 10^− 4^	0.409	0.001	0.275	Ns	–	0.001	0.289	Ns	–
Negative	845.675	801.897	[236] [M]-	3.22 × 10^− 4^	0.303	5.00 × 10^−5^	0.428	Ns	–	4.82 × 10^− 5^	0.289	Ns	–
Negative	846.678	801.89	[236] [M + 1]-	1.99 × 10^− 4^	0.289	5.00 × 10^− 5^	0.440	Ns	–	4.44 × 10^− 5^	0.437	Ns	–
Negative	859.691	817.445	[258] [M]-	2.61 × 10^− 4^	0.255	1.06 × 10^− 4^	0.425	Ns	–	1.85 × 10^− 4^	0.394	Ns	–
Negative	860.694	817.445	[258] [M + 1]-	0.001	0.253	1.06 × 10^−4^	0.422	Ns	–	1.85 × 10^− 4^	0.397	Ns	–
Negative	861.587	636.5	[260] [M]-	0.001	0.313	Ns	–	Ns	–	Ns	–	Ns	–
Negative	897.631	662.634	[299] [M]-	0.001	0.332	Ns	–	0.028	−0.228	Ns	–	Ns	–
Negative	899.647	783.472	[301] [M]-	2.62 × 10^−4^	0.279	Ns	–	Ns	–	0.004	0.297	Ns	–
Positive	339.289	796.101		0.002	−0.382	Ns	–	1.85 × 10^−15^	0.721	Ns	–	0.031	0.341
Positive	612.556	793.586		0.004	−0.382	Ns	–	2.41 × 10^−12^	0.663	Ns	–	0.022	0.347
Positive	616.499	695.687	[70] [M + 1]+	0.004	−0.384	Ns	–	1.37 × 10^−17^	0.757	Ns	–	Ns	–
Positive	759.637	657.349	[160] [M]+	0.006	0.290	1.06 × 10^−4^	0.427	Ns	–	2.93 × 10^−4^	0.396	Ns	–
Positive	760.64	657.377	[160] [M + 1]+	0.002	0.342	6.45 × 10^−4^	0.362	Ns	–	6.49 × 10^− 4^	0.362	Ns	–
Positive	761.644	657.307	[160] [M + 2]+	2.79 × 10^−4^	0.378	1.65 × 10^− 4^	0.392	0.028	−0.247	4.06 × 10^− 4^	0.369	Ns	–
Positive	774.656	745.664	[184] [M + 1]+	0.003	0.360	0.001	0.309	Ns	–	5.13 × 10^−4^	0.350	Ns	–
Positive	781.619	657.212	[192] [M]+	0.001	0.330	2.43 × 10^−4^	0.386	Ns	–	3.04 × 10^− 4^	0.375	Ns	–
Positive	782.622	657.224	[192] [M + 1]+	3.53 × 10^−4^	0.380	1.06 × 10^− 4^	0.417	Ns	–	2.99 × 10^− 4^	0.384	Ns	–
Positive	783.625	657.209	[192] [M + 2]+	0.001	0.353	0.008	0.284	Ns	–	0.018	0.258	Ns	–
Positive	785.653	680.778	[202] [M]+	2.61 × 10^−4^	0.357	0.002	0.313	0.028	−0.246	0.004	0.286	Ns	–
Positive	786.656	680.694	[202] [M + 1]+	2.37 × 10^−4^	0.369	0.002	0.317	0.028	−0.231	0.003	0.293	Ns	–
Positive	788.671	792.889	[204] [M + 1]+	0.001	0.235	2.59 × 10^−4^	0.389	Ns	–	7.01 × 10^− 4^	0.345	Ns	–
Positive	800.672	771.37	[226][M + 1]+	0.001	0.335	0.001	0.306	Ns	–	0.003	0.291	Ns	–
Positive	801.684	809.306	[229] [M]+	1.99 × 10^−4^	0.349	1.06 × 10^− 4^	0.387	Ns	–	2.99 × 10^− 4^	0.358	Ns	–
Positive	802.687	809.268	[229][M + 1]+	1.99 × 10^−4^	0.348	7.58 × 10^−5^	0.417	Ns	–	1.71 × 10^− 4^	0.400	Ns	–
Positive	807.634	680.491	[239] [M]+	9.83 × 10^−4^	0.362	Ns	–	Ns	–	Ns	–	Ns	–
Positive	815.699	824.707	[257] [M]+	2.61 × 10^−4^	0.251	0.001	0.345	Ns	–	0.004	0.297	Ns	–
Positive	816.703	824.708	[257] [M + 1]+	2.61 × 10^−4^	0.252	0.001	0.353	Ns	–	0.003	0.304	Ns	–

**Fig. 2:**
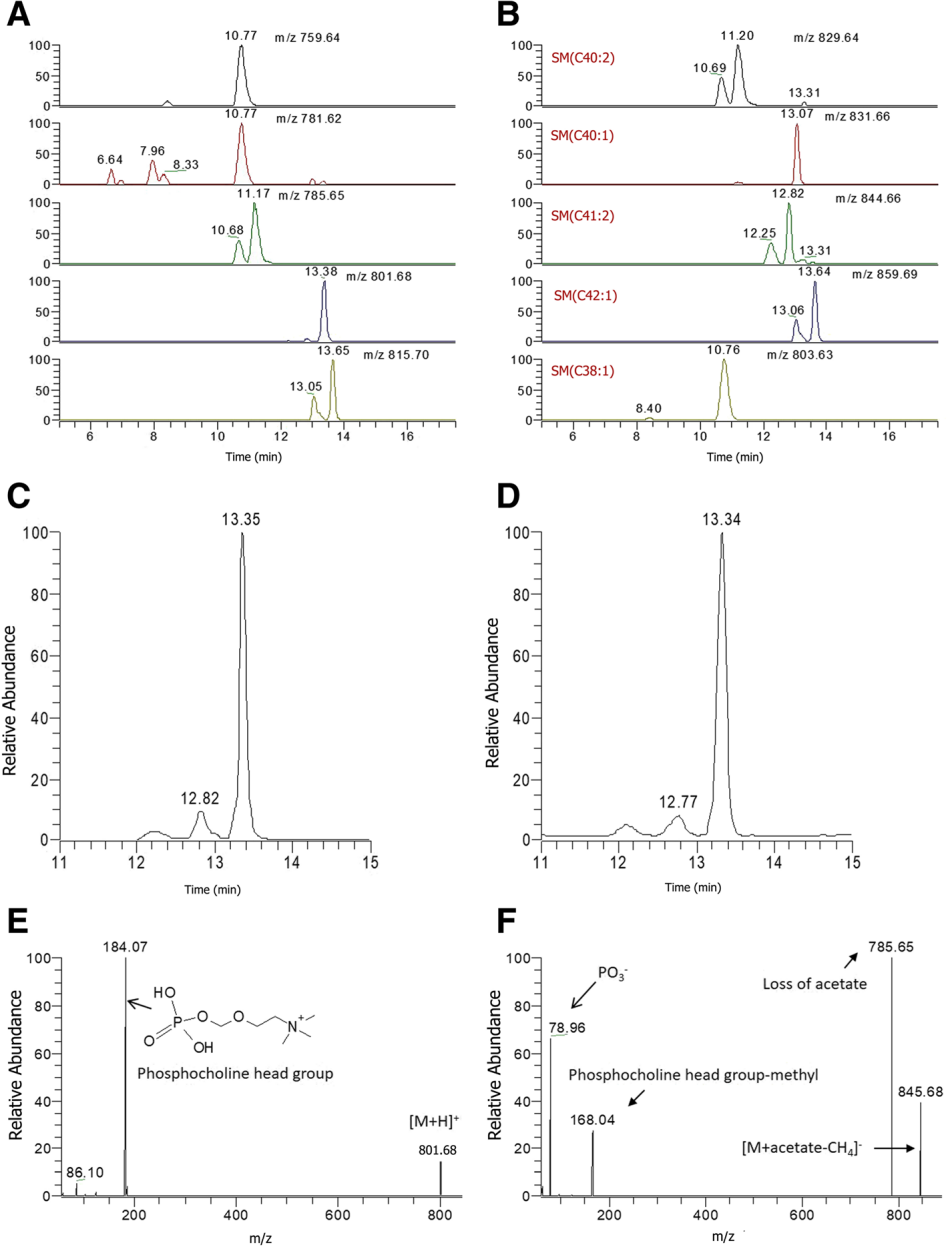
Identification of metabolites associated with plasma HDL cholesterol. Extracted ion chromatograms of the unknown compounds significantly associated with HDL cholesterol were obtained by liquid chromatography mass spectrometry (UPLC-MS) using a CSH C18 column. Data are shown for profiles acquired in positive (**a**) and negative (**b**) ionization mode. Illustrations of extracted ion chromatograms of an unknown compound in positive (**c**) and negative ion mode (**d**) and their corresponding MS/MS (**e**, **f**) at a respective collision energy of 20 and 30 (arbitrary unit)

In contrast, only eight spectral features that associated with HDL were also associated with serum triglycerides, and only two features were associated with FBS (Table 2; Additional file 2: Table S1). Analyses of correlations between CAD and lipidomic data did not identify lipid features associated with CAD (Data not shown). Peak annotation of the features significantly associated with HDL cholesterol using the CAMERA tool identified ions originating from the same compound and their adduct/isotope/in-source fragment formations. In particular, significantly associated compounds were characterized by one or several isotopic peaks in both negative and positive ionization mode (Table 2). A total of 34 features were detected in both ionization modes (Table 2; Fig. 2a and b). These features were mostly concentrated in the *m/z* region between 750 and 810, which is the domain of phospholipids and sphingomyelins. Fourteen features (in that domain) were significantly associated with plasma HDL cholesterol levels, total and LDL cholesterol.

### Structural assignment and metabolite identification

There was no apparent matching between the set of features in positive and negative ion modes suggesting that the metabolites had undergone loss of specific fragments. Each compound was characterized by its *m/z* and retention time (Table 2). A same compound can be ionized in positive and negative ion mode, respectively forming a [M + H] + ion and [M + HCOO]- adduct. For instance, *m/z* 759.64 in positive ionization mode and 803.63 in negative ionization mode stand for the same compound. In the positive ion mode, the fragmentation experiment highlighted a common fragmentation patterns for all these compounds (Fig. 2c-f). They both generate the *m/z* 184.0739 fragment-ion corresponding to the phosphocholine head group. At this stage, compounds could be glycerophospholipids or sphingolipids. The parity of the ions is one of the features of the type of lipids. Phosphatidylcholines (PC), Phosphatidylethanolamines (PE) and phosphatidylserines (PS) have odd *m/z*, whereas sphingomyelins (SM) and phosphatidyl inositols (PI) have even *m/z* for their [M + H]^+^ ion. Negative ion mode fragmentation gave complementary information to positive ion mode (Fig. 2c-f). In this mode, the [M + acetate-CH_4_]- ion gives the most informative fragmentation pathway with specific ion fragments, thus allowing the identification of the lipid class. However, even though the lipid class can be identified, databases still provide various candidate compounds corresponding to the same *m/z*.

## Discussion

We report results from a metabolome-wide association study (MWAS) based on untargeted lipidomic profiling that identified correlations between lipid features and lipoprotein metabolism, which contribute to CAD risk. We demonstrate association of lipoproteins (Total, HDL and LDL cholesterol) with largely independent series of metabolic features. Additional analyses of metabolic features underlined the associations between HDL cholesterol and sphingomyelins.

Our untargeted lipidomic profiling strategy relies on simultaneous quantitative analysis of a large number of metabolite features that can be tested for association with any phenotype recorded in the cohort, including disease conditions and CAD endophenotypes. This approach, which was coined metabolome-wide association study (MWAS) [12], has been successfully used to demonstrate correlative relationships between disease and metabolites, including for example between hypertension and urinary formate [12] and the dicarboxylic acid hexadecanedioate [20]. When extended to multiple phenotypes in the context of untargeted analysis of spectral data, MWAS provides detailed information on coordinately regulated metabolites (metabotypes) by bringing together compounds that are not necessarily involved in the same metabolic process but respond to the same biological stimulus or pathophysiological condition. Our findings indicate that lipid features associated with serum HDL cholesterol were largely independent from those associated with serum triglycerides. Conversely, each of these phenotypes exhibited specific patterns of correlations with lipid features. Mostly, the features exhibited a positive correlation with the HDL, LDL, total cholesterol and FBS levels, but a negative correlation for TGs. Interestingly, only three metabolites didn’t follow this pattern of correlation and correlated negatively with HDL and positively with TGs levels, two of these features correlated positively with FBS levels. The three metabolites (shown in Table 2) are acquired in positive ionization mode, and present respectively an *mz* of 339.289, 612.556 and 616.499. The metabolites presenting an *mz* of 339.289 and 612.556 correlated positively with FBS levels. The metabolite corresponding to an *mz* of 612.556 is diacylglycerol (DAG), the two remaining metabolites are of unknown nature.

MWAS deepens the characterization of metabolite biomarkers associated with phenotypes and enables integration of information from individual compounds into biological pathways. This is illustrated by the conserved correlative relationships of plasma HDL, LDL and total cholesterol with the same set of lipid features, which we were able to annotate as sphingomyelins. Sphingomyelins map to the pathway of sphingolipid metabolism. They consist of over 18 molecular species, which are products of phosphocholine derived from phosphatidylcholine and ceramide [21]. Sphingomyelinase and sphingomyelin synthase are responsible for the interconversion of sphingomyelins and ceramide. Sphingolipids play a significant physiological role in maintaining cell wall integrity, cell growth, cell differentiation and critical signal transduction pathways, which have recently attracted considerable interest as potential therapeutic targets in cardiovascular diseases.

Association of sphingolipids with lipoproteins, which are major risk factors of CAD, is anticipated in our study, where elevated plasma sphingomyelins as well as the ratios of aortic and plasma ceramide are associated with increased risk of cardiovascular diseases [22–24]. Sphingolipids promote lipoprotein aggregation, plaque instability, inflammation and apoptosis [25]. Moreover, the concentration of sphingomyelins in the artery wall increases with aging and comprises 70–80% of the phospholipids in atherosclerotic lesions [25]. Of note, atherosclerotic plaques exhibit increased levels of ceramide and sphingomyelins, which contribute to inflammation of coronary artery smooth muscle cells. This role might be particularly significant in the context of the metabolic syndrome, where overabundance of long-chain saturated fatty acids stimulates sphingolipid synthesis and turnover in the liver, which leads to the generation of sphingolipid-enriched lipoproteins, facilitating plaque development.

Low level of high-density lipoprotein cholesterol (HDL-C) and elevated triglyceride/HDL-C ratio are important risk factors for cardiovascular disease in the Lebanese population. Similar to our study, sphingomyelins, the most abundant sphingolipid in lipoproteins, are directly correlated to lipoproteins, where approximately 63–75% and 25–35% of sphingomyelin is associated with LDL and HDL, respectively in healthy subjects. We provide evidence of association of sphingomyelin and lipoproteins among subjects with established CAD. These associations stem from an intricate relationship between sphingolipids and lipoproteins on the cellular, molecular and biochemical levels.

First, sphingomyelinis the major sphingolipid in HDL particles, and the content of sphingomyelin affects the metabolic function of HDL. Specifically, the proportion of sphingomyelin in HDL predicts the capacity of serum HDL particles to accept cellular cholesterol, known as reverse cholesterol transport, since sphingomyelin is positively correlated with its fractional efflux. In fact, plasma enzymes involved in HDL metabolism such as lecithin–cholesterol acyltransferase (LCAT) or phospholipid transfer protein are inhibited by HDL sphingomyelin content, which negatively impacts reverse cholesterol transport. Additionally, over-expression of sphingomyelin synthase increases the atherogenic potential of lipoproteins. Among all HDL-related parameters, sphingomyelin showed the strongest evidence of association with the presence of CAD and number of coronary stenosis, as shown in a multivariate analysis of data from women with angiographically assessed disease, highlighting the role of reduced cholesterol efflux capacity as an important factor accounting for the inverse association between HDL-cholesterol and CAD. It has been shown that sphingolipids alter LDL functions in tissues, where LDL-associated sphingomyelins affect their aggregation and accumulation in macrophages as well as LDL kinetics. This is achieved through the action of sphingomyelinase in coronary plaques, transforming sphingomyelin into ceramide, which aids in LDL aggregation and uptake.

Second, experimental, observational and genetic studies have shown that sphingolipids are involved in the cellular regulation of lipoprotein synthesis by regulating the post-transcriptional activation of sterol regulatory element-binding proteins (SREBPs), which are key transcription factors of lipid synthetic genes. For instance, SREBP-2 activates the LDL receptor and all genes required for cholesterol synthesis. Hence, increased sphingolipid synthesis in the liver is associated with increased cholesterol synthesis, whereas inhibition of sphingomyelin synthesis by myriocin is associated with downregulation of SREBP-regulated genes, inhibition of cholesterol and triglyceride synthesis and decrease in atherosclerotic plaque [26, 27]. Inhibition of sphingolipid synthesis, however, also induces the expression of apolipoprotein A1 and LCAT, leading to increased plasma HDL as a counter-regulatory, anti-atherogenic particle, which is in line with our finding that sphingomyelin directly correlates with HDL concentration. Besides sphingomyelins, ceramides also affect HDL function by increasing plasma membrane expression of ATP-binding cassette receptor A1 (ABCA-1), which is crucial for formation of nascent HDL and correlates positively with plasma HDL. Hence, by regulating lipoprotein synthesis and function, sphingolipids are major risk factors of CAD.

In conclusion, our data underline the power of untargeted lipidomic profiling for systematic quantitative profiling of series of metabolites simultaneously, to uncover correlative relationships between disease status and endophenotypes and metabolic/metabotype biomarkers, which represent observable end-points of altered regulation of biochemical pathways. The complexity of sphingolipid species, described using new lipidomic methodologies, and their distribution in different lipoprotein particles under different experimental conditions are promising avenues for further research. Multiple targets in the sphingolipid metabolism pathway provide ample opportunities for drug discovery. Overall, this has the potential to contribute to improving our knowledge of disease risk and to characterizing therapeutic targets and advanced disease preventive approaches.

## Additional files

Additional file 1: **Figure S1.** Representative total ion current (TIC) chromatogram profiles in positive (A) & negative (B) ion modes. Data were obtained following untargeted liquid chromatography mass spectrometry (LC-MS) analysis with a CSH C18 columnof a quality control samples representing a pool of plasma samples. Retention times (X-axis) are plotted against relative intensity of the spectral peaks (Y-axis). Approximate positions of the families of lipid compounds in the chromatogram are shown: *CE* Ceramides, *DG* Diacyglycerol, *FA* Fatty Acids, *PC* Phosphatidylcholines, *PE* Phosphatidylethanolamines, *PG* Phosphatidylglycerolipids, *PI* phosphatidylinositols, *PS* phosphatidylserines, *SM* sphingomyelins, *TG* Triacylglycerol. **Figure S2.** 3-D Principal component analysis of mass spectrometry data in the cohort. Metabolome data from plasma samples of the cohort processed with a CSH C18 column were analyzed after filtering and normalization for the positive mode (A) and the negative mode (B). **Figure S3.** Metabolome-wide association between metabolic features and plasma LDL (A, B) and total (C, D) cholesterol. Metabolomic data were obtained by liquid chromatography mass spectrometry (LC-MS) with a CSH C18 column.Spectral features referenced by their mass to charge ratio (X-axis) are plotted against the statistical significance of the association to plasma LDL and total cholesterol (Y-axis). Association results are shown for data in negative (A, C) and positive (B, D) ionization modes. (PPTX 579 kb)

Additional file 2: **Table S1.** Details of metabolic features significantly associated (FDR adjusted *P* < 0.05) with quantitative variations ofHDL cholesterol, and relationships with other phenotypes analysed in the study cohort. Correlations statistics of metabolic features are given. m/z, mass to charge ratio. rt., retention time. (XLSX 195 kb)
