# Supplementary material for: Untargeted Mass Spectrometry Lipidomics identifies correlation between serum sphingomyelins and plasma cholesterol
Source: Lipids Health Dis. 2019 Feb 2;18:38. doi: 10.1186/s12944-018-0948-5 (PMC6359757; doi:10.1186/s12944-018-0948-5)
Supplement: Supplementary file 1 — Figure S1. Representative total ion current (TIC) chromatogram profiles in positive (A) & negative (B) ion modes. Data were obtained following untargeted liquid chromatography mass spectrometry (LC-MS) analysis with a CSH C18 columnof a quality control samples representing a pool of plasma samples. Retention times (X-axis) are plotted against relative intensity of the spectral peaks (Y-axis). Approximate positions of the families of lipid compounds in the chromatogram are shown: CE Ceramides, DG Diacyglycerol, FA Fatty Acids, PC Phosphatidylcholines, PE Phosphatidylethanolamines, PG Phosphatidylglycerolipids, PI phosphatidylinositols, PS phosphatidylserines, SM sphingomyelins, TG Triacylglycerol. Figure S2. 3-D Principal component analysis of mass spectrometry data in the cohort. Metabolome data from plasma samples of the cohort processed with a CSH C18 column were analyzed after filtering and normalization for the positive mode (A) and the negative mode (B). Figure S3. Metabolome-wide association between metabolic features and plasma LDL (A, B) and total (C, D) cholesterol. Metabolomic data were obtained by liquid chromatography mass spectrometry (LC-MS) with a CSH C18 column.Spectral features referenced by their mass to charge ratio (X-axis) are plotted against the statistical significance of the association to plasma LDL and total cholesterol (Y-axis). Association results are shown for data in negative (A, C) and positive (B, D) ionization modes. (PPTX 579 kb) [file 12944_2018_948_MOESM1_ESM.pptx]

## Slide 1
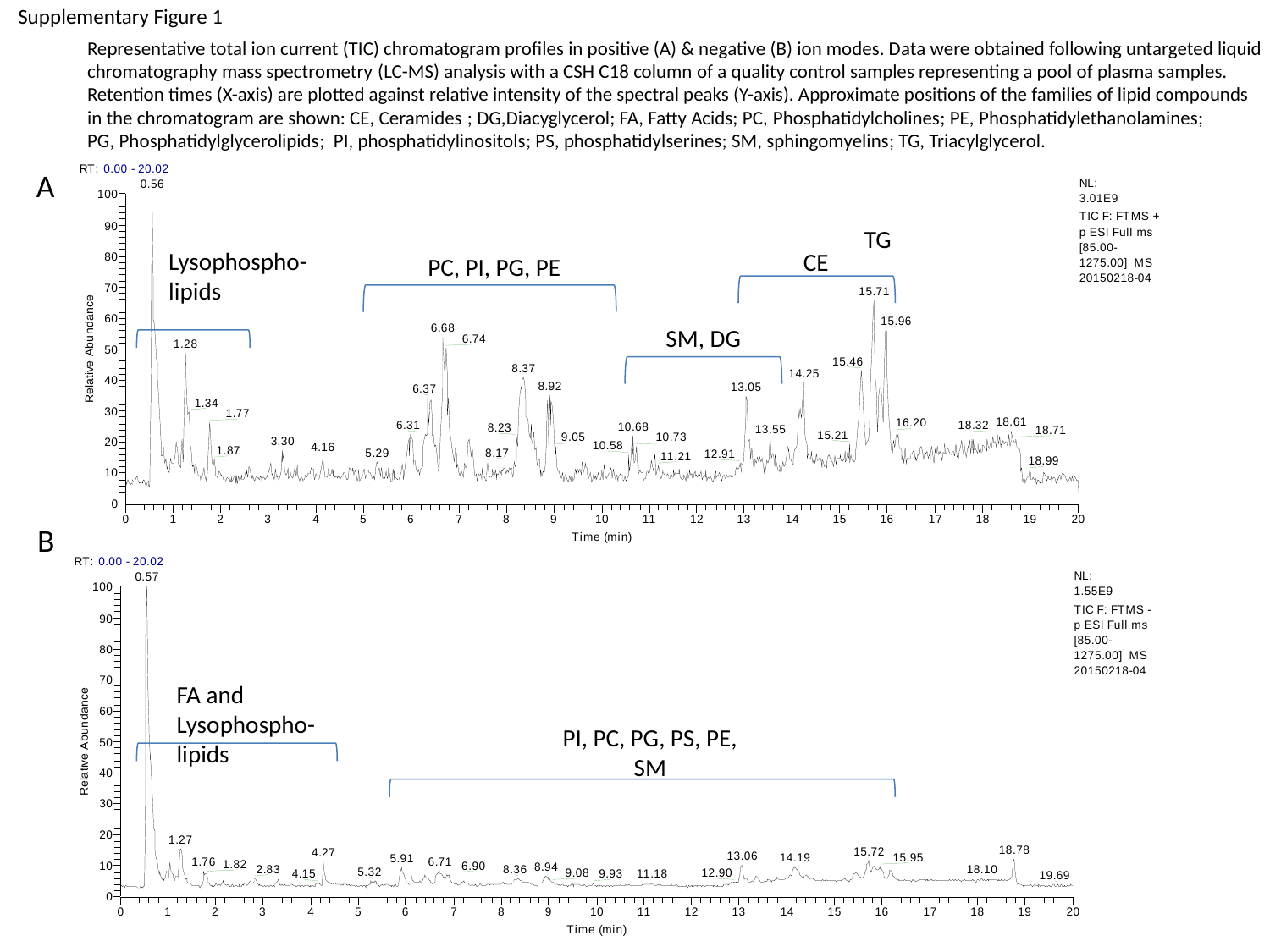

Supplementary Figure 1
Representative total ion current (TIC) chromatogram profiles in positive (A) & negative (B) ion modes. Data were obtained following untargeted liquid chromatography mass spectrometry (LC-MS) analysis with a CSH C18 column of a quality control samples representing a pool of plasma samples. Retention times (X-axis) are plotted against relative intensity of the spectral peaks (Y-axis). Approximate positions of the families of lipid compounds in the chromatogram are shown: CE, Ceramides ; DG,Diacyglycerol; FA, Fatty Acids; PC, Phosphatidylcholines; PE, Phosphatidylethanolamines; PG, Phosphatidylglycerolipids; PI, phosphatidylinositols; PS, phosphatidylserines; SM, sphingomyelins; TG, Triacylglycerol.
A
TG
Lysophospho-
lipids
CE
PC, PI, PG, PE
SM, DG
B
FA and Lysophospho-
lipids
PI, PC, PG, PS, PE, SM

## Slide 2
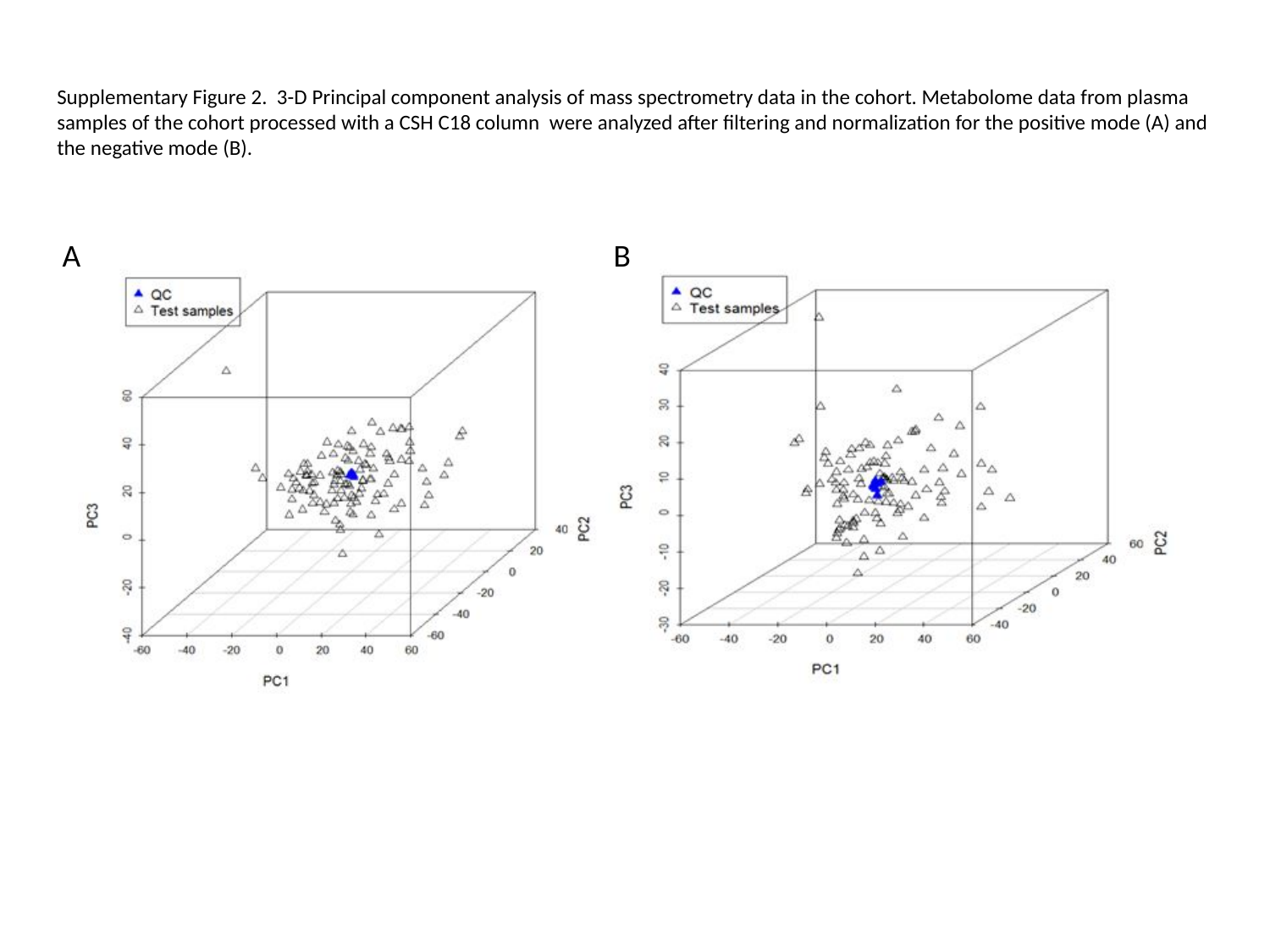

Supplementary Figure 2. 3-D Principal component analysis of mass spectrometry data in the cohort. Metabolome data from plasma samples of the cohort processed with a CSH C18 column were analyzed after filtering and normalization for the positive mode (A) and the negative mode (B).
A
B

## Slide 3
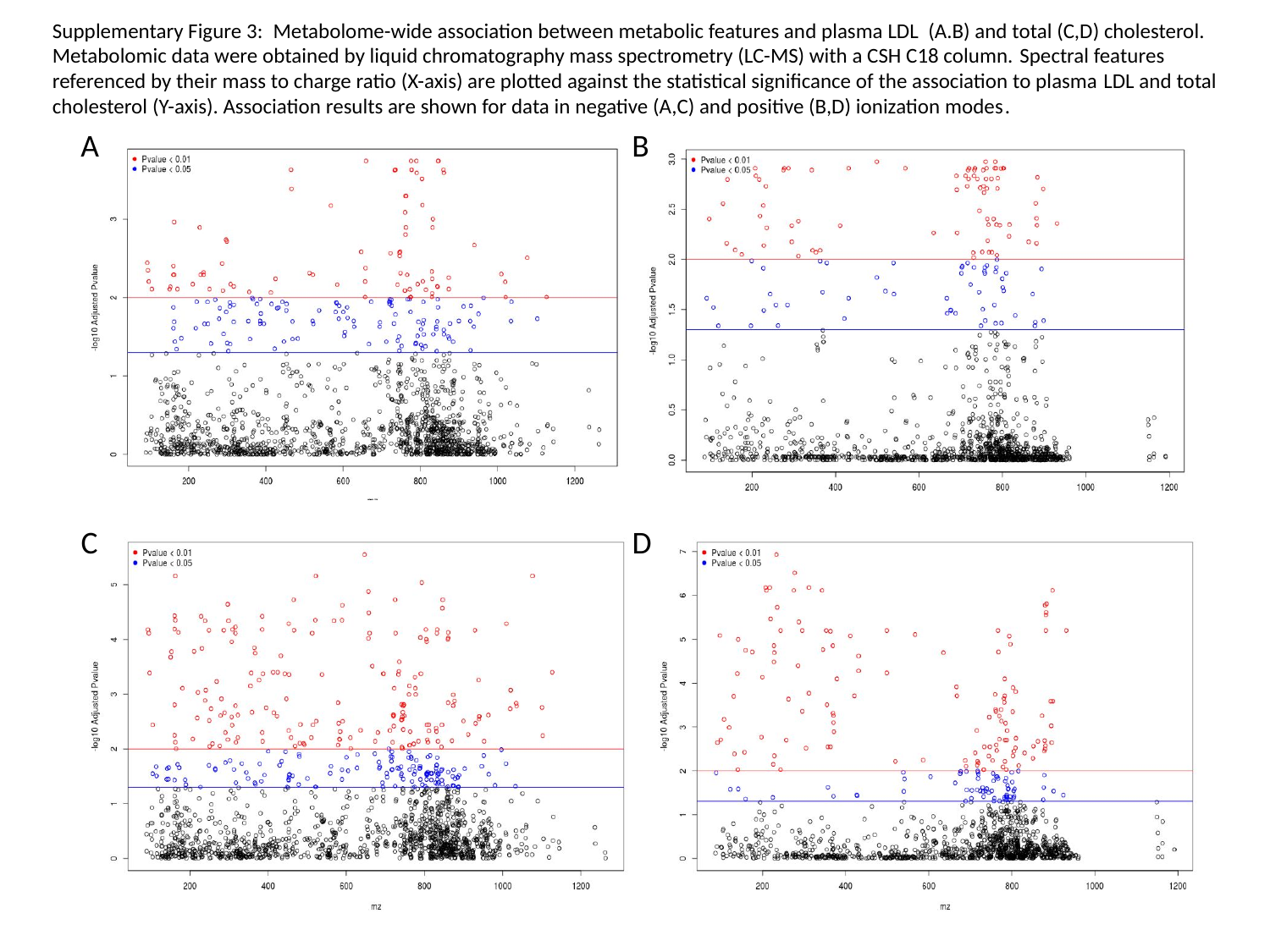

Supplementary Figure 3: Metabolome-wide association between metabolic features and plasma LDL (A.B) and total (C,D) cholesterol. Metabolomic data were obtained by liquid chromatography mass spectrometry (LC-MS) with a CSH C18 column. Spectral features referenced by their mass to charge ratio (X-axis) are plotted against the statistical significance of the association to plasma LDL and total cholesterol (Y-axis). Association results are shown for data in negative (A,C) and positive (B,D) ionization modes.
A
B
C
D
